# The Association Between Polycystic Ovary Syndrome and Its Dermatological Manifestations

**DOI:** 10.7759/cureus.6855

**Published:** 2020-02-03

**Authors:** Amanpreet Kaur Sekhon, Azka Shahid Zergham, Gantuya Tserenpil, Amal Mebasher, Bilal Haider Malik

**Affiliations:** 1 Internal Medicine, California Institute of Behavioral Neurosciences and Psychology, Fairfield, USA

**Keywords:** metabolic syndrome, acne, pcos, hirsutism, hyperandrogenism, dermato-endocrinology

## Abstract

Polycystic ovary syndrome (PCOS) commonly occurs in reproductive-age females. It elevates the hormonal levels, creates an imbalance in the metabolic system, and affects their reproductive system too. A number of studies have been conducted on PCOS, and it has been diagnosed together with several dermatological conditions. In this paper, we attempt a traditional review to study the relationship between PCOS and its cutaneous manifestations the patients are predisposed to. To uncover this association, we gathered information from English-language articles on the Pubmed database using six keywords. Materials were also collected from studies done on animal models, which helped in putting down all the data together and interlinking them with other studies. From this data collection, it is ultimately concluded that the association between PCOS and dermatological conditions is a very intricate interconnecting network comprising many factors, such as inflammation, genetics, and hormonal. This study raises some questions that are still unanswered. We believe further research is needed to uncover the various facts about this disease and its associations, in order to make its management more effective. As there is a strong association between PCOS and certain dermatological disorders, it is recommended to develop a questionnaire that should be distributed to every woman who presents to dermatology departments with symptoms that are linked to PCOS, as it will help in diagnosing the condition at an early stage.

## Introduction and background

Polycystic ovary syndrome (PCOS), also known as Stein-Leventhal syndrome, affects 6-10% women of fertile age group [1]. It is the most prevalent heterogeneous condition, with cardinal features consisting of hyperandrogenism and reproductive and metabolic dysfunction. Among these features, hyperandrogenism is the most crucial clinical finding with different symptoms of presentation [2].

PCOS is mainly diagnosed based on hyperandrogenism, anovulation, and polycystic ovarian morphology. The four phenotypes of PCOS are as follows:

· Presence of hyperandrogenism, oligomenorrhea, and polycystic ovary

· Hyperandrogenism and oligomenorrhea

· Oligomenorrhea and polycystic ovary

· Hyperandrogenism and polycystic ovary [3]

PCOS presents with a wide spectrum of common dermatological manifestations, such as hirsutism, acne, seborrheic dermatitis (SD or seborrhea), and androgenetic alopecia (AGA). These conditions can present alone or alongside other features of virilization. The four conditions mentioned above are tetrad of seborrhoea, acne, hirsutism, and alopecia (the SAHA syndrome) [4]. PCOS has been associated with acanthosis nigricans; pyoderma gangrenosum, acne, and hidradenitis suppurativa (PASH) syndrome; acrochordons, *Demodex folliculorum*, striae, xanthoma, and psoriasis. An additional two cases with mucosal pigmentation suggesting Peutz-Jegher syndrome have also been reported to have an association with PCOS [5-8].

Dermato-endocrinology is a part of medicine that focuses on numerous fascinating facets of disorders that occur due to disturbance to the regulatory mechanism of the skin. It also focuses on the prevention and treatment of these disorders [9]. The etiology of PCOS is multifactorial, with a clear genetic component exhibited by the familial aggregation studies with epidemiological illustrations. Epigenetic changes are reported in literature describing the hypothesis of environmental factors reprogramming the future ovarian function [10]. The genetic part is determined by the gene variants, epigenetics, race or ethnicity, and the environmental element, which further includes diet, lifestyle, and socioeconomic, toxicologic, and geographical factors [11]. PCOS etiology is not completely understood. The common presentations are the primary focus of management to avoid long-term sequelae such as insulin resistance, cardiovascular events, oncological risks, infertility, depression, and poor quality of life, which can lead to further diverse consequences [12].

PCOS can have drastic economic effects. Approximately four billion dollars are spent every year in the United States for the screening of the disease and the treatment of its various manifestations, including hirsutism, infertility, and diabetes mellitus. The Australian health system invests more than 800 million dollars annually in the treatment of this disease [13]. Therefore, gaining a better understanding of the various aspects of this disease is of utmost importance, so that we can diagnose this at an earlier stage and prevent future comorbidities.

In this review, we summarize the most relevant and recent reports related to PCOS and its related dermatological conditions. We also briefly address its genetic and hormonal components. We emphasize the association between PCOS and various cutaneous manifestations to gain a comprehensive understanding of this disorder.

## Review

Method

A comprehensive search was carried out on Pubmed to conduct a detailed study on the association between PCOS and its various dermatological manifestations; all the data was collected after a thorough analysis of the literature. A large number of articles were found using regular and Medical Subject Headings (MeSH) keywords. Preferred Reporting Items for Systematic Review and Meta-analysis (PRISMA) guidelines, quality assessment tools, and statistical analysis were not used as this was a traditional review article. PCOS study data was collected from research among the female population, but one study among males was considered as it revealed the existence of male PCOS, which presents as AGA. The whole data was collected in a legal and ethical manner. The keywords used for the search and the articles they yielded are presented here (Table 1).

**Table 1:** Keywords used for literature search PCOS: polycystic ovary syndrome

Keywords	Database	Number of articles returned
Metabolic syndrome	Pubmed	79,596
Acne	Pubmed	17,908
PCOS	Pubmed	10,730
Hirsutism	Pubmed	6,379
Hyperandrogenism	Pubmed	4,476
Dermato-endocrinology	Pubmed	297

Results

This traditional review article search was accomplished by using six keywords. A search with metabolic syndrome showed 79,596 studies; acne returned 17,908 results; PCOS showed 10,730 results; hirsutism yielded 6,379 studies; hyperandrogenism fetched 4,476 studies; dermato-endocrinology yielded 297 studies. A search that contained the terms “PCOS, Dermatology” together returned 104 articles. MeSH keyword acne yielded 11 articles; hyperandrogenism fetched four studies; hirsutism yielded two studies.

Dermato-endocrinology studies linking PCOS and its cutaneous manifestations

Any unsettling pathophysiological influence on metabolic adjustments in the body may offer clarification for the cutaneous signs of the disease [14]. The accentuation on dermatological appearances in PCOS is exceptionally crucial for early detection of PCOS as it aids in preventing the disease from advancing to its long-term sequelae [6,15]. For instance, in a study conducted by Botchkarev VA, it was concluded that bone morphogenetic protein (BMP) is included in the homeostasis of skin epidermis and development of hair follicles, interceding its activity through a mitogen-activated protein (MAP) kinase pathway, with a prenatal and postnatal impact [16]. In another study carried out on BMP, the signaling protein was found expressed in granulosa cells of ovaries ordinarily. It was affirmed that the levels of certain BMPs were high in females with PCOS, showing its association in regenerative abnormalities [17]. Hence, there is evidence of BMP presence in both the studies, raising the following question: is BMP responsible for the cutaneous manifestations in PCOS women or can it be utilized as an indicator marker? Immunology, genetic susceptibility, and irregular hormonal levels through various metabolic pathways are found in PCOS pathogenesis pertaining to the dermatology field.

Dermatoimmunological association

Inflammation is one of the underlying fundamental mechanisms found common in PCOS and its different dermatological manifestations. This produces a connection pertaining to immuno-dermatology and immune-metabolism, demonstrating that they are closely connected to each other [18]. Various inflammatory proteins related to PCOS are tumor necrosis factor-alpha (TNFα), interleukin-6 (IL-6), interleukin-18 (IL-18), and C-reactive protein (CRP) [19].

To confirm this and to assemble more evidence, a hormonal and histopathological study was done on PCOS rats using pentoxifylline, a drug with antioxidant and anti-tumor necrosing factor-alpha properties. A solid association was indeed found between ovarian function modification and immune markers, uncovering the part played by chronic inflammation and oxidative stress pathways in PCOS [20].

The nuclear receptor subfamily 4 group A member 1 (NR4A1) gene belongs to the family of orphan nuclear receptors, which plays a role in the molecular mechanisms by expressing in ovaries of PCOS patients and is upregulated by androgens and transcription factors [21]. It was concluded from a study conducted by Murphy EP and Crean D that NR4A1 is required in inflammatory conditions as this suppresses the inflammatory markers and settles the response of immune cells. As PCOS patients generally have hyperandrogenism, NR4A1 overexpresses in PCOS [22]. This information led us to raise another query: does NR4A1 level increases because of the inflammatory mechanism as a triggering factor or due to hyperandrogenism in PCOS or both?

SD is an inflammatory condition that presents as erythematous skin with yellow-colored scales along with greasy look in sebaceous areas because of the increased discharge of sebum by sebaceous glands, which is increased by androgens. Several studies have been conducted to uncover the profundity of its molecular mechanisms. In one study, a great number of immune cells were identified, such as interleukin-17 (IL-17), CD45+ leukocytes, CD5+ lymphocytes, γⴃT cells in connection to it and ZNF750/MPZL3 pathway, which plays a critical part in the pathogenesis of SD [23,24]. Additionally, this inflammatory aggregation in SD disrupts the skin barrier called the epidermis and further produces an immune reaction to *Malassezia* yeasts [25].

Acne is an inflammation of pilosebaceous glands with numerous pathogenic variables as it is a neuroendocrine organ. In PCOS, it can be affected by abundance dihydrotestosterone levels as well as an insulin-like growth factor (IGF-1) [26].

Hidradenitis suppurativa is an apocrine gland inflammation with follicular impediment. A study was done regarding hidradenitis suppurativa and comorbidities associated with it, of which PCOS was second-most prevalent. The main etiology behind hidradenitis suppurativa is insulin resistance and inflammation [27,28].

Very few studies conducted to date have revealed an association between PCOS and psoriasis. In a study done by Moro F et al., psoriatic patients were compared to healthy patients in terms of predisposition to PCOS, and a significant correlation was found between both. The psoriatic patients were found more prone to get PCOS in comparison with the control group [29]. In psoriasis, keratinocytes play an important role in inflammatory mechanisms in the skin by producing cytokines-TNF, interferons, IL-17, and IL-20 members, which further leads to the generation of pro-inflammatory cytokines such as IL-1, IL-6, and TNF-α [30].

Genetics of PCOS and its dermatological manifestations

To investigate the gene expression in PCOS, differentially expressed genes (DEGs) were examined undergoing methylation through abnormal biological pathways of lipid metabolism and steroidogenesis within the granulosa cells of PCOS, demonstrating the pathogenesis of PCOS [31]. PCOS is not only a disease of females but inherited by males as well because of its genetic transmission characteristic. The most common presentation in males is the early onset of AGA, which is also dermatology-related [32].

A study was done by Smith KJ and Germain M on two known cases of PCOS patients presenting with mucosal pigmentation, suggesting Peutz-Jegher syndrome probably due to the abnormal functioning of serine/threonine-protein kinase 4/liver kinase B1 (STK4/LKB1) protein [8]. In another study, detailed research was carried on LKB1. It was observed that LKB1 has an inhibitory action on androgens in theca cells of the ovary, whereas the excess amount of androgens suppresses LKB1 activity [33]. This suggests a possible LKB1 link to PCOS disease. Now the question emerges as to whether PCOS presence leads to Peutz-Jegher or vice-versa; in other words, is hyperandrogenism inhibiting LKB1 by feedback mechanism or is LKB1 inhibition presence in Peutz-Jegher syndrome failing to suppress androgen levels, leading to PCOS?

Insulin-like growth factor receptor (IGFR) is responsible for insulin resistance and high androgen levels; hence, the absence of IGFR implies low testosterone and no insulin resistance. IGFR is understood to have a negative impact on CYP17 and a positive one on CYP19. Thus, in PCOS, where androgen is on a higher level, there will be IGFR activity present and, consequently, high CYP19 and dysfunctioning CYP17 levels are recorded [33,34]. In a study among 198 Iranian patients diagnosed with acne vulgaris conducted to shed some light on the association between acne vulgaris and two genotypes of CYP 450 (CYP 17 and CYP 19), it was proved that both genotypes are connected to the occurrence of acne vulgaris [35].

Hormones

In a hormonal study of PCOS by Gowri BV et al., six dermatological conditions were studied correlating with hormonal levels, which were observed to be high in PCOS patients. The study was conducted on patients who were fasting, and insulin levels were found to be higher compared to other hormones In 67.5% of them; testosterone levels were found to be elevated in 55% of the patients [6].

In women with PCOS, due to the excess production of androgens by ovaries and adrenals, 5α reductase activity increases peripherally [36]. This is understood to be related to acne, AGA, and hirsutism. Furthermore, 5α reductase leads to the development of insulin resistance [37]. PCOS is found to be composed of a complex chain reaction in which hyperandrogenism can be exacerbated by weight gain and insulin resistance, leading to the progression of PCOS [2].

Hirsutism is defined as excessive thick-hair growth in facial and body regions. It is an early manifestation of virilization and correlates closely with elevated testosterone production [37]. Increased action of 5α reductase leading to more formation of active metabolites of testosterone, particularly in hair follicles, was noted in some studies [38,39].

PCOS disorder comprises a spectrum of cutaneous manifestations and *Demodex folliculorum* is noted to be more predominant in women with PCOS women [7]. It has been reported in some studies that acne vulgaris and *Demodex folliculorum* are interconnected to each other. However, the related pathological mechanism is still unclear [40].

## Conclusions

The main motive of this study was to explore the link between PCOS and its cutaneous manifestations pertaining to the dermato-endocrinology field. We attempted to highlight the strong link connecting dermatology and endocrinology via immunology mainly, and also by other factors, revealing multiple possible pathways that ultimately led to cutaneous manifestations in PCOS. Skin manifestations mainly depend on the phenotype of PCOS, and they end up becoming causative factors for each other, which further leads to skin signs and symptoms. There are particular skin disorders reported in patients with PCOS, but further research is needed to delve deep into them and uncover the related molecular mechanisms, which may help in the early detection and better management of PCOS.
